# Adenovirus Infections in African Humans and Wild Non-Human Primates: Great Diversity and Cross-Species Transmission

**DOI:** 10.3390/v12060657

**Published:** 2020-06-18

**Authors:** Hacène Medkour, Inestin Amona, Jean Akiana, Bernard Davoust, Idir Bitam, Anthony Levasseur, Mamadou Lamine Tall, Georges Diatta, Cheikh Sokhna, Raquel Adriana Hernandez-Aguilar, Amanda Barciela, Slim Gorsane, Bernard La Scola, Didier Raoult, Florence Fenollar, Oleg Mediannikov

**Affiliations:** 1 IHU Méditerranée Infection, 13385 Marseille CEDEX 05, France; hacenevet1990@yahoo.fr (H.M.); amoninestin@gmail.com (I.A.); bernard.davoust@gmail.com (B.D.); anthony.levasseur@univ-amu.fr (A.L.); laminetall30@gmail.com (M.L.T.); cheikh.sokhna@ird.fr (C.S.); bernard.la-scola@univ-amu.fr (B.L.S.); didier.raoult@gmail.com (D.R.); florence.fenollar@univ-amu.fr (F.F.); 2 Aix-Marseille University, IRD, AP-HM, Microbes, MEPHI, 13385 Marseille CEDEX 05, France; 3 PADESCA Laboratory, Veterinary Science Institute, University Constantine 1, El Khroub 25100, Algeria; 4 Aix-Marseille University, IRD, AP-HM, SSA, VITROME, 13385 Marseille CEDEX 05, France; idirbitam@gmail.com (I.B.); georges.diatta@ird.fr (G.D.); 5 Faculté des Sciences et Techniques, Université Marien NGOUABI, Brazzaville, Democratic Republic of the Congo; 6 Laboratoire National de Santé Publique, Brazzaville, Democratic Republic of the Congo; jakiana2000@yahoo.fr; 7 Superior School of Food Sciences and Food Industries, Algiers 16004, Algeria; 8 VITROME IRD 198, Campus IRD/UCAD, Hann les Maristes, Dakar, Senegal; 9 Department of Social Psychology and Quantitative Psychology, Faculty of Psychology, University of Barcelona, Passeig de la Vall d’Hebron 171, 08035 Barcelona, Spain; r.a.hernandez-aguilar@ibv.uio.no; 10 Jane Goodall Institute Spain and Senegal, Dindefelo Biological Station, Dindefelo, Kedougou, Senegal; amanda.b@janegoodall.es; 11 Direction Interarmées du Service de Santé des Armées des Forces Françaises Stationnées à Djibouti; slim.gorsane@intradef.gouv.fr

**Keywords:** adenoviruses, non-human primates, humans, prevalence, Africa, zoonotic diseases, cross-species transmission

## Abstract

Non-human primates (NHPs) are known hosts for adenoviruses (AdVs), so there is the possibility of the zoonotic or cross-species transmission of AdVs. As with humans, AdV infections in animals can cause diseases that range from asymptomatic to fatal. The aim of this study was to investigate the occurrence and diversity of AdVs in: (i) fecal samples of apes and monkeys from different African countries (Republic of Congo, Senegal, Djibouti and Algeria), (ii) stool of humans living near gorillas in the Republic of Congo, in order to explore the potential zoonotic risks. Samples were screened by real-time and standard PCRs, followed by the sequencing of the partial DNA polymerase gene in order to identify the AdV species. The prevalence was 3.3 folds higher in NHPs than in humans. More than 1/3 (35.8%) of the NHPs and 1/10 (10.5%) of the humans excreted AdVs in their feces. The positive rate was high in great apes (46%), with a maximum of 54.2% in chimpanzees (*Pan troglodytes*) and 35.9% in gorillas (*Gorilla gorilla*), followed by monkeys (25.6%), with 27.5% in Barbary macaques (*Macaca sylvanus*) and 23.1% in baboons (seven *Papio papio* and six *Papio hamadryas*). No green monkeys (*Chlorocebus sabaeus*) were found to be positive for AdVs. The AdVs detected in NHPs were members of *Human mastadenovirus E* (HAdV-E), HAdV-C or HAdV-B, and those in the humans belonged to HAdV-C or HAdV-D. HAdV-C members were detected in both gorillas and humans, with evidence of zoonotic transmission since phylogenetic analysis revealed that gorilla AdVs belonging to HAdV-C were genetically identical to strains detected in humans who had been living around gorillas, and, inversely, a HAdV-C member HAdV type was detected in gorillas. This confirms the gorilla-to-human transmission of adenovirus. which has been reported previously. In addition, HAdV-E members, the most often detected here, are widely distributed among NHP species regardless of their origin, i.e., HAdV-E members seem to lack host specificity. Virus isolation was successful from a human sample and the strain of the Mbo024 genome, of 35 kb, that was identified as belonging to HAdV-D, exhibited close identity to HAdV-D members for all genes. This study provides information on the AdVs that infect African NHPs and the human populations living nearby, with an evident zoonotic transmission. It is likely that AdVs crossed the species barrier between different NHP species (especially HAdV-E members), between NHPs and humans (especially HAdV-C), but also between humans, NHPs and other animal species.

## 1. Introduction

Adenoviruses (AdVs), members of the *Adenoviridae* family, are DNA viruses that naturally infect many vertebrates, including humans and non-human primates (NHPs). Their name derives from their initial isolation from human adenoids in 1953 [1]. Since then, human adenoviruses (HAdVs) have been increasingly recognized as major contributors to clinical illness, from mild respiratory infections in young children (known as the common cold) to life-threatening multi-organic diseases in people with weakened immune systems. It is estimated that more than 90% of the human population is seropositive for at least one serotype of AdVs [2,3]. Illnesses include upper and lower respiratory tract diseases, conjunctivitis, acute hemorrhagic cystitis, meningoencephalitis, diarrhea, intussusceptions, celiac disease, hepatitis, myocarditis and obesity, with certain clinical diseases associated with specific adenoviral species and genotypes [3]. Despite their role as pathogens, some HAdVs and simian adenoviruses (SAdVs) have been used or proposed as tools for vaccine delivery and gene therapy [4].

Until now, within the genus *Mastadenovirus* (including all human adenoviruses), 103 human AdV genotypes have been assigned in high resolution using genomics (http://hadvwg.gmu.edu/). They have been divided into seven distinct species (*Human mastadenovirus A* to *Human mastadenovirus G*, informally HAdV-A to HAdV-G) that were originally distinguished by their biological, clinical and restriction enzyme digestion properties and were reconfirmed using omics data. AdVs within the genus *Mastadenovirus* could infect other mammalian hosts, such as NHPs, bats, bovines, canines, deer, dolphins, equines, murines, ovines, swine, sea lions, skunks, squirrels and tree shrews [3]. Novel human and animal AdVs continue to be identified and characterized [5,6]. In addition, there are AdVs in four other genera within the *Adenoviridae* family that infect animals. These are *Atadenovirus*, type species: *Ovine atadenovirus D*; *Aviadenovirus,* type species: *Fowl aviadenovirus A*; *Ichtadenovirus*, type species: *Sturgeon ichtadenovirus A*; and *Siadenovirus*, type species: *Frog siadenovirus A* (https://talk.ictvonline.org/taxonomy/). As with humans, AdV infections in animals can cause diseases that range from asymptomatic to fatal [3].

For millennia, interactions between humans and their closest living relatives have led to an inherent risk of pathogen transfer. NHPs are increasingly implicated as potential sources of emerging zoonotic diseases in humans [7,8]. Indigenous groups that depend on wildlife for survival were exposed to the risk of the transmission of NHP pathogens through hunting, consumption of bushmeat [9] and through other ways; for example, by sharing non-flowing water sources, fruits and plant sources that NHPs have used. Concerning adenoviruses, virologists have long wondered about the possibility that NHP adenoviruses may one day pose a risk to humans [10]. Although adenoviruses are generally considered to be rather host species-specific viruses, canine adenovirus 1 has a wider host range and can infect members of the *Canidae, Mustelidae* and *Ursidae* families [11]. The host range and zoonotic potential of simian adenoviruses has more recently become an area of interest [10,12]. 

Simian adenoviruses (SAdVs) have been described in macaques (SAdV-1 to SAdV-15), African green monkeys (SAdV-16 to SAdV-18 and SAdV-20), baboons (SAdV-19) and chimpanzees (SAdV-21 to SAdV-25) [13]. Many more simian AdVs have been described, like New World monkey and prosimian AdVs [14]. In 2009, Roy S et al. isolated and characterized 30 novel great ape AdVs from the feces of chimpanzees, bonobos and gorillas, as well as three macaque AdVs. All of them were captive NHPs, held in facilities and zoos in North America. Virus isolation was performed, and complete genomes were sequenced and tentatively named SAdV-25.2 to SAdV-50 [13]. Most SAdVs have proven to be very similar to HAdVs. Currently, the naming of a species follows the principle that if an adenovirus species with primate hosts contains at least one type of HAdV, the species is called a HAdV species, otherwise it is called a SAdV species. As a result, most SAdVs have been grouped correspondingly into the HAdV- B, C, E and G species [13,15,16,17,18,19,20,21].

In the present study, we sought to investigate the presence and molecular diversity of AdVs in wild African NHPs, including great apes (gorillas and chimpanzees), macaques and other monkeys (baboons, green monkeys), living in close proximity to or outside human settlements. In addition, we assessed the AdV infections of a local human population from the Republic of Congo, who were living near gorillas, in order to evaluate potential zoonotic transmissions. Fecal samples were collected and tested by PCR/sequencing, and the adenoviruses were typed based on their DNA polymerase partial gene. A human strain was isolated by viral cell culture and described. 

## 2. Materials and Methods

### 2.1. Animals and Study Area

In Senegal, in August 2016, 48 fecal samples of western chimpanzees (*Pan troglodytes verus*) were collected from three sites located within the Dindefelo Community Natural Reserve in the Kédougou region. Site 1: three degraded stool samples (12°22′57.1404″ N, 12°17′16.7172″ W), Site 2: seven degraded stool samples (12°22′53.1732″ N, 12°17′26.7936″ W) and Site 3: thirty-eight fresh stool samples (12°22′47.7084″ N, 12°17′48.588″ W). In 2015, green monkeys (*Chlorocebus sabaeus*) and Guinea baboons (*Papio papio*) were sampled in the Niokolo-Koba National Park (NKNP), a World Heritage Site and one of the oldest natural protected areas in Africa. The NKNP is situated in Southeastern Senegal and borders with Guinea. Fresh feces from 4 green monkeys and 7 baboons, which were habituated individuals, were collected near the Niokolo forestry guardhouse of the NKNP (13°04′28.6″ N 12°43′18.2″ W). The study was approved by the Senegalese Ministry of the Environment (Direction of the National Parks, No. 1302, 16 October, 2015). The Direction des Eaux, Forêts, Chasses et Conservation des Sols of the Republic of Senegal gave authorization to collect and export feces samples (No. 1914/DEF/DGF of 5 June 2016).

In the Republic of Congo, gorilla (*Gorilla gorilla*) feces were collected from the Lésio-Louna (LLR) and South-West Léfini (SWLR) gorilla reserves (2°58′33.1″ S 15°28′33.4″ E), as part of a collaborative project between the Government of the Republic of Congo and the Aspinall Foundation, which manages a protected area of 170,000 ha located about 140 km north of Brazzaville. About 35 gorillas inhabit the Lesio-Louna/Léfini Natural Reserves and, in August 2015, we collected 16 non-fresh fecal samples. The project was approved by the Ministry of Health (No 208/MSP/CAB.15 of 20 August 2015) and the Forest Economy and Sustainable Development (No 94/MEFDD/CAB/DGACFAP-DTS of 24 August 2015) of the Republic of Congo. Moreover, between 2017 and 2019, 12 other gorilla feces samples from the LLR, ten samples from Odzala-Kokoua National Park (OKNP) (1.3206° N, 14.8455° E) and one sample from Nouabale-Ndoki National Park (NNNP) (2.5857° N, 16.6291° E) were collected. For NHPs, fresh fecal samples were collected at sleeping sites, feeding sites and places where the primates had been observed. In addition, in 2017, human stool samples were collected after obtaining the verbal consent of all the participants because of their low level of literacy. A total of 38 samples were collected, including 35 from the local population of the village of Mbomo, located inside the OKNP, and 3 from eco-guards in the LLR. All great ape samples were stored in absolute alcohol, and fresh samples and human samples were first stored at −20 °C before being sent from the Republic of Congo to France for analysis. 

In the Republic of Djibouti, 6 fecal samples of hamadryas baboons (*Papio hamadryas*) were collected in 2017. These baboons lived outside the village of Oueah, 38 km from the city of Djibouti (11°29′56.1″ N 42°51′14.8″ E). This collection was carried out in partnership with the Center for Studies and Research of Djibouti.

In Algeria, fecal samples were collected from 69 Barbary macaques (*Macaca sylvanus*), with the authorization of the management of the Chréa National Park (CNP), including 30 samples collected from two sites, the Stream of Monkeys and the Gorges of la Chiffa in Blida Province, 50 km north of Algiers (36°23′42.9″ N 2°45′53.6″ E), and 39 samples from Cap Carbon (36°46′31.6″ N 5°06′11.2″ E) in the suburbs of Béjaïa, 250 km east of Algiers. These primates were synanthropic and lived in close contact with the people who provided them with food.

No experimentation was conducted on any of the NHPs in our study as all fecal samples were collected from the ground. The sampling was non-invasive and did not disrupt any wild animal.

All humans in our study were apparently healthy. In addition, the fact that the NHPs’ feces were not diarrheic may perhaps be some indication of them also being healthy. All collected samples were taken to the IHU Méditérranée Infection Laboratory, 13005 Marseille, France, for analysis. They were identified and stored at either −20 °C or −80 °C.

### 2.2. DNA Extraction

Viral DNA extraction was performed using the Qiagen Virus Mini Kit^®^ v2.0 (Qiagen, Courtaboeuf, France) on BIOROBOT EZ1 (Qiagen, Courtaboeuf, France), according to the manufacturer’s instructions. Firstly, about 40 g of each stool was mixed with 360 µL of G2 buffer and 40 µL of proteinase K (Qiagen, Courtaboeuf, France). This was subjected to a mechanical lysis with tungsten beads (Qiagen, Courtaboeuf, France), using a FastPrep-24TM 5G Grinder, before being incubated overnight at 56 °C. Then, viral DNA was extracted from 190 µL of supernatant with 10 µL of internal control (Enterobacteria phage T4). DNA was eluted in 130 µL volume, then aliquoted in individual PCR tubes to an amount of 50 µL of pure extracted DNA. Then, other aliquots of 50 µL of DNA were diluted to 1:10, and finally, one-third of 50 µL of DNA diluted to 1: 100. DNA tubes were stored at −20 °C until use.

The DNA extraction and dilutions were controlled by qPCR, targeting the phage T4 (Table 1). Based on the results of the qPCR for extraction control, DNA which had been diluted to 1/10th was chosen for adenovirus testing.

### 2.3. Real-Time PCR Assays (qPCR) 

First, all samples were screened using qPCR targeting the T4 phages [22], and after that they were screened by qPCR targeting a conserved region of the hexon gene of all 51 HAdV prototypes [23]. The qPCR amplifications were performed in a CFX96 real-time system (Bio-rad Laboratories, Foster City, CA, USA). Reactions were carried out in a final volume of 20 µL, containing 5 µL of DNA template, 10 µL of Master Mix Roche (Eurogentec, Seraing, Belgium), 0.5 µL each primer per reaction at a concentration of 20 µM, 0.5 µL UDG and 0.5 µL of each probe at a concentration of 5 µM. The TaqMan cycling conditions included two hold steps at 50 °C for 2 min, followed by 95 °C for 15 min and 40 cycles of two steps each (95 °C for 30 s and 60 °C for 30 s). The PCR systems used for the study are detailed in Table 1. Each PCR plate contained 96 wells. DNA from the cultured adenovirus was included as a positive control and master mixtures as negative controls in each test. 

### 2.4. Genetic Amplification by Standard PCR, Sequencing and Phylogeny

All samples were tested by a sensitive and specific nested PCR (Table 1), using outer primers in the first reaction and inner primers in the second. The nested PCR was used for the amplification of the DNA polymerase partial gene and the specific diagnostic for adenoviral shedding [13].

For gene amplification, PCR reactions were performed in a total volume of 50 µL, consisting of 25 µL of AmpliTaq Gold master mix, 18 µL of ultra-purified water DNAse-RNAse free, 1 µL of each primer (20 µM of concentration) and 5 µL of the DNA template. In the second amplification, we used only 1 µL of PCR1 product rather than 5 µL of DNA and 22 µL of ultra-purified water rather than 18 µL. The thermal cycling conditions for both PCR amplifications were as follows: incubation step at 95 °C for 15 min, 40 cycles (only 30 cycles for the second PCR) of one minute at 95 °C, 30 s for the annealing at 54 °C, 1.5 min (30 s for the second PCR) of elongation time at 72 °C. Finally, we included an extension step for five minutes at 72 °C. 

PCR amplification was performed in a Peltier PTC-200 model thermal cycler (MJ Research Inc., Watertown, MA, USA). The results of amplification were visualized by electrophoresis on 2% agarose gel. Samples that had Ct < 35 in qPCR and a good band of nested PCR were selected for sequencing. Amplicons were purified using NucleoFast 96 PCR plates (Macherey Nagel EURL, Hoerdt, France), as per the manufacturer’s instructions, and sequenced using the Big Dye Terminator Cycle Sequencing Kit (Perkin Elmer Applied Biosystems, Foster City, CA, USA) with an ABI automated sequencer (Applied Biosystems). The obtained electropherograms were assembled and edited using ChromasPro software (ChromasPro 1.7, Technelysium Pty Ltd., Tewantin, Australia) and compared with those available in the GenBank database compiled by NCBI BLAST (https://blast.ncbi.nlm.nih.gov/Blast.cgi). The sequences obtained were aligned and compared with each other and with those of the AdVs available in the GenBank database. A maximum likelihood method was used to infer the phylogenetic analyses and tree reconstruction was performed using MEGA software version 7 (https://www.megasoftware.net/). Bootstrap analyses were conducted using 1000 replicates.

### 2.5. Cultivation and Virus Isolation

Fecal samples from positive NHPs and humans for the molecular detection of AdVs were subjected for virus isolation in cell cultures. The large particles were removed by low speed centrifugation, and the supernatant was filtered through 0.2 µm-pore sized syringe filters. A volume of 100 µL of each filtered sample was inoculated into HEp2 and MRC5 cells which had been grown in a MEM medium with 10% fetal bovine serum (FBS) for 14 days. Cultures were observed every 2 days to detect the appearance of a cytopathogenic effect (CPE). qPCR targeting adenovirus was performed at day 0 and day 14 in each culture to confirm that CPE was related to adenovirus growth. Virus isolates collected from the cell lines were analyzed using the molecular approaches described above.

The isolated viruses were first analyzed using the same molecular approaches described above and then subjected to next generation sequencing (NGS) for the sequencing of the whole adenovirus genome.

### 2.6. Genome Sequencing 

Whole adenovirus genome analysis was performed using DNA extracted from the culture isolate using next generation sequencing (NGS). The genomic DNA (gDNA) of AdV Mbo024 was quantified by a Qubit assay with the high sensitivity kit (Life technologies, Carlsbad, CA, USA) to 0.2 ng/µL. The genomic DNA was then sequenced with the MiSeq Technology (Illumina Inc., San Diego, CA, USA) with the paired end strategy and was barcoded in order to be mixed respectively with 11 other genomic projects prepared with the Nextera XT DNA sample prep kit (Illumina).

To prepare the paired end library, a dilution was performed so that 1 ng of each genome was needed as input to prepare the paired end library. The tagmentation step fragmented and tagged the DNA. Then, limited cycle PCR amplification (12 cycles) completed the tag adapters and introduced dual-index barcodes. After purification on AMPure XP beads (Beckman Coulter Inc., Fullerton, CA, USA), the libraries were normalized on specific beads according to the Nextera XT protocol (Illumina). Normalized libraries were pooled into a single library for sequencing on the MiSeq. The pooled single strand library was loaded onto the reagent cartridge and then onto the instrument, along with the flow cell. Automated cluster generation and paired end sequencing with dual index reads were performed in a single 39-h run in 2x250-bp.

Total information of 54,397 Gb was obtained from a 559 K/mm^2^ cluster density with a cluster passing quality control filters of 97.1%. Within this run, the index representation for AdV Mbo024 was determined to index 4.8722. The 1,083,204 paired end reads were filtered according to the read qualities.

### 2.7. Bioinformatics Analysis

The quality of the sequence data obtained was controlled using the CLC Genomics Workbench v7 (Qiagen, https://www.qiagenbioinformatics.com/products/clc-genomics-workbench/) and the assembly was performed by SPADES V3.13.0 [24]. Sequences were trimmed with MiSeq and Trimmomatic [25] software, whereas untrimmed data were processed only using MiSeq software. To reduce assembly deviations, we used GapCloser software [26]. Scaffolds that had a nucleotide number <800 (bp) and scaffolds that had a depth value lower than 25% of the mean depths were removed. The best assembly was selected using different criteria (number of scaffolds, N50, number of N) [27]. After assembly, we applied a mapping program between our genome and that of the reference genomes, human adenovirus 25 (JN226752) and human adenovirus 13 (JN226747), to produce consensus sequences in order to ensure the consistency of the methodology using a scaffold builder [27] with a minimum identity for merging contigs (80%), minimum length for ambiguously mapped contigs (95%) and maximum gap length allowed (5000 nt) (Supp. Material S1). Finally, the genes (E1A, E1B, IX, IVa2, E2A, E2B, pTP, L1-L5, E3, E4, U-exon) were analyzed using a homology search engine, the local BLAST [28,29]. The sequences were aligned using MUSCLE with default parameters.

## 3. Results

The DNA extraction was validated by the qPCR targeting the T4 phage: All samples tested positive (Ct ≤ 35). Ct values were (26.73–37) mean: 30.16 ± 1.96 for pure DNAs and (25–35.03) mean: 29.36 ± 2.21 for DNAs diluted at 1:10. Ct values for DNA dilutions at 1:100 were higher than the others or even completely negative for some samples. Given these results, DNAs diluted at 1:10 were chosen for the adenoviruses study.

**Figure 1:**
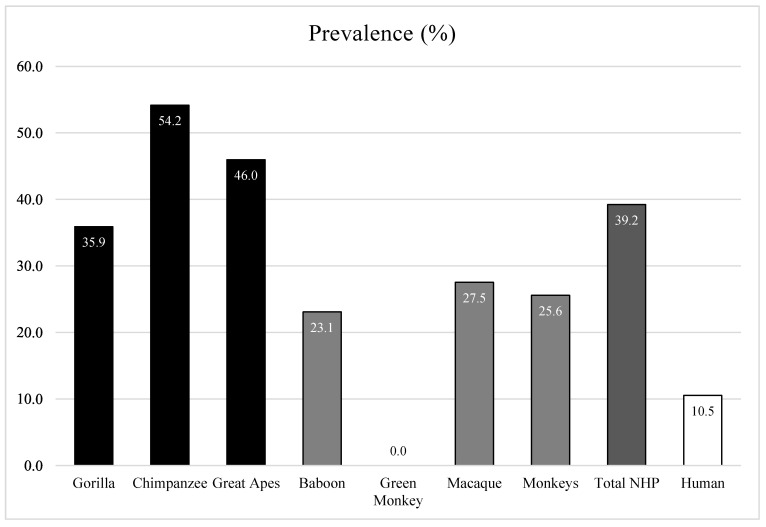
Prevalence of adenoviruses in African humans and NHPs. **Deep dark**: Great apes, including gorillas from the Republic of Congo and chimpanzees from Senegal. **Gray**: Monkeys, including Guinea baboons from Senegal, hamadryas from Djibouti and Algerian Barbary macaques from Algeria. **White**: humans from the Republic of Congo, living in contact with gorillas.

Based on the results of qPCR (Ct < 35) and standard PCR simultaneously (presence of the specific band), the total prevalence of adenoviruses was significantly different (z test, *p*-value = 0.0001) between African NHPs, at 35.8% (62/173), and humans from the Republic of Congo, at 10.5% (4/38). Adenovirus carriage was different according to the species of NHP (Khi2 test, *p*-value = 0.014). Monkeys were less infected, at 25.6% (22/86), than great apes, at 46% (40/87) (z test, *p*-value = 0.007). The prevalence rates were as follows: 27.5% (19/69) in macaques, 23.1% (3/13) in baboons, 0% in green monkeys (0/4), 35.9% (14/39) in gorillas and 54.2% (26/48) in chimpanzees. In the Republic of Congo, where we collected gorilla and human samples simultaneously, AdVs were more frequent in gorillas (35.9%) than humans (10.5%) (4/38) (z test, *p*-value < 0.0001) (Figure 1, Table 2).

**Figure 2:**
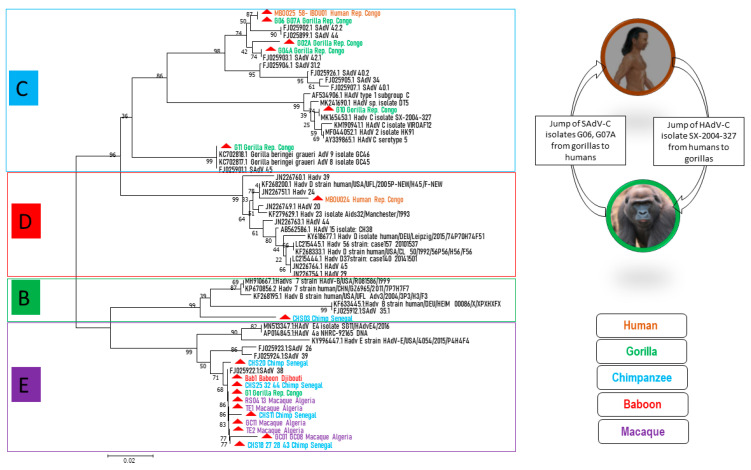
Molecular phylogenetic analysis by maximum likelihood method based on a short (250 bps) sequence alignment of the DNA polymerase gene of human and simian adenoviruses. The evolutionary history was inferred using the maximum likelihood method, based on the Tamura three-parameter model. Sequences were aligned by the ClustalW method and compared with each other as well as with available DNA polymerase sequences from GenBank. Sequences in this study are indicated by the following colors: **brown** for humans; **green** for gorillas; **blue** for chimpanzees; **violet** for macaques and red for baboons. Sequences from GenBank are in **black**. AdV types from HAdV-C, -B and -E were detected in African NHPs and from HAdV-C and -D in humans. HAdV-C jumped from humans to gorillas and, inversely, HAdV-C strains G06 and G07A jumped from gorillas to humans from the Republic of Congo. The tree with the highest log likelihood (−1102.33) is shown. The percentage of trees in which the associated taxa clustered together is shown next to the branches. The tree is drawn to scale, with branch lengths measured in the number of substitutions per site. The analysis involved 59 nucleotide sequences. The confidence probability (multiplied by 100) for the inside length of the branch is greater than 0, as estimated by the bootstrap test (1000), which is shown next to the branches.

We succeeded in obtaining 25 sequences of ≈250 bps of adenoviral DNA polymerase genes from the stool of NHPs: six sequences from gorillas, ten from chimpanzees, seven from macaques and one from baboons from Djibouti. Four sequences were obtained from human stool samples from the Republic of Congo. The sequences were compared with each other and with the DNA polymerase sequences available in the GenBank database (Table S1). Seven sequences from chimpanzees, one from gorillas, one from baboons and seven from macaques exhibited at least a 97% identity with each other and a 99% identity with SAdV-38 (FJ025922/HB426671). They clustered together and with HAdV-**E** members from GenBank (Figure 2). One sequence from a chimpanzee showed a 97% identity with SAdV-41.2 (FJ025927) and grouped with HAdV-**B** types. One human sequence was 98% identical to a HAdV-**D** type (KF268200). Three human and two gorilla sequences were perfectly similar to each other and two other sequences from gorillas showed >97% identity with them. All these sequences clustered together with species HAdV-**C** (Figure 2).

In the Republic of Congo, we found HAdV-C and E members in gorillas and HAdV-C and D members in humans, with an abundance of C members in both humans and gorillas (Table S1). In Senegal, HAdV-B and E members were detected in chimpanzees, and the E members were the most prevalent ones. AdV-E was also found in baboons from Djibouti. In Algeria, the macaques were infected by AdV-E types. In this study, the green monkeys were negative for AdVs, probably due to the limited number of samples (only four samples).

Viral isolation was successful in HEp2 cells inoculated with a human stool sample with 20 Ct in the qPCR control at day 14. No virus was isolated from the NHP stool samples. The Mbo024 strain of the whole genome of a 35 kb length showed a 97.6% identity with HAdV- 25 (JN226752) and a 96.4% identity with HAdV- 13 (JN226747) from HAdV-D detected in a human from the United States of America. The fifteen recognized genes for AdVs were identified in the Mbo024 strain and compared to the reference AdV species; they were close to the HAdV-D types (Table S2). The HAdV- Mbo024 whole genome was deposited in the GenBank database under accession number (LR796137.1).

## 4. Discussion

Since the recurring influenza scares (H5N1 since 1997 and H7N9 in 2013) and the recent coronavirus outbreaks (SARS-CoV in 2003, MERS-CoV in 2010 and SARS-CoV-2 in 2019), the topic of zoonotic viruses, particularly viruses that can cross the interspecies barrier from their natural host and eventually cause disease in humans, has aroused great interest. For any virus, adaptation to a new host must meet several basic requirements and key events. In this study, we detected several species of AdVs in African great apes (gorillas and chimpanzees) and monkeys (baboons and Algerian macaques), as well as in local people residing near gorillas in the Republic of Congo. Interestingly, the positive rate was 3.3 folds higher in NHPs than in humans. More than one third of the NHPs and one tenth of the humans in this study carried and excreted AdVs in their feces. The high prevalence of AdVs in NHPs could be explained by the fact that these animals are repeatedly infected, although these infections would have to be prolonged and frequent to maintain virus circulation and increase their prevalence. There was much less virus excretion in human feces, which may have been due to a more efficient T-cell response to viruses [30].

AdV infections have been reported at a high prevalence in wild gorilla and chimpanzee populations, as well as in other great apes [5,13,31,32,33,34]. In the present study, the AdV-infection rate in gorillas from the Republic of Congo was 35.9%, which is close to the previously reported rates in free ranging gorillas in the same area (44.9%) [31] or those from Loango National Park, Gabon (48%) [31]. Captive gorillas in zoos had a much higher prevalence of shedding (36%–100%) [13]. The results in our Senegalese chimpanzee study population (54.2%) were higher than in the Cameroon/Democratic Republic of Congo study (40%) [13] and in another population of savannah-dwelling chimpanzees from the Issa Valley in Tanzania (37%) [35], and they were lower than the reported rate of 68% in the Republic of Congo [31]. These differences seem to be marginal and could be due to differences in sample collection and conservation conditions, study region, seasonality or degree of anthropogenic disturbance of the habitat, but they are less likely to be due to laboratory differences, since all the studies used the same PCR assay [13]. Our results confirm the persistence of AdV feces shedding and circulation in African great apes. In addition, the occurrence of AdVs in macaque feces ranged from 13% to 100%: 13% in Oregon—rhesus, 67% Covance—rhesus, 88% GTP—cynomolgus and 100% GTP—rhesus [13]. For the first time, we detected the DNA of AdVs in 27% of Barbary macaques from Algeria (27%). It was also reported that African monkeys could be infected with AdVs [5]. We found a positive rate of 17% in the sub-Saharan monkeys of our study, including 23% in baboons (from Senegal and Djibouti) and 0% in green monkeys. Although this difference in results between the macaques in North Africa and the other monkeys in sub-Saharan Africa was not statistically significant and was related to the difference in sample sizes (69 macaques versus only 13 baboons and 4 green monkeys), the difference was not statistically significant. In addition, macaques already live in contact with tourists, which may be the cause of transmission between the two species. Although prevalence statistics are presented here, the reality may be underestimated due to the potential deterioration of the sample. Great apes seemed to be more infected (46%) than monkeys (25.6%) and humans (10.5%). On the one hand, apes could be repeatedly infected and, consequently, viruses may be maintained and circulated fluently. On the other hand, the human T-cell response to the viruses is more effective, leading to the limitation of virus shedding.

Our study revealed a great diversity of AdVs among African humans and NHPs. We identified 25 AdV sequences from NHPs and four from humans. From NHPs, based on the 250 bp of AdV DNA polymerase gene [13], we detected the members of three AdV species: these were principally HAdV-E in 72% of cases, followed by HAdV-C in 24% of cases and HAdV-B in 4% of cases. These AdVs have been previously reported [5,31,32,34,36]. In addition, it could be possible, because of the methodology, that very divergent segments were not picked up by the primers, especially those of monkey AdVs. Therefore, the non-identified AdVs here could be SAdVs or AdVs from other species which were not amplified by the applied primers. Furthermore, the DNA polymerase fragment was selected in order to be highly conserved. It is therefore no surprise that it was found to be considerably conserved.

As demonstrated in Figure 2, HAdV-E members seemed to be well adapted in NHPs, since they were present in all NHPs in this study, except green monkeys (perhaps because of the small number of samples), and they clustered both with one another and with SAdV-E members. Although AdVs are generally considered to be rather host species-specific viruses, there are some exceptions [3]. Indeed, HAdV-E could be an exception, since African great apes, as well as macaques or other monkeys in our study, carried HAdV-E members. For HAdV-E type occurrence in this study, wild Senegalese chimpanzees were the most numerous hosts (50% of cases), followed by macaques (39%) and then gorillas and baboons (5.5% of cases for each). Herein, the hypothesis that NHP AdV members of the HAdV-E originated from chimpanzees [32,33], and because chimpanzees, gorillas and baboons all live in sub-Saharan Africa, we can suppose that the gorilla and baboon HAdV-E types of this study were the result of chimpanzee AdV transmission. The evolution of HAdV-E viruses and their adaptation to new hosts requires further investigation.

HAdV-C types were detected in the gorilla feces samples from the LLR and OKNP and eco-guards as well as in humans living around the OKNP. These AdV-C members consist of three clades: (i) Clade 1: one gorilla isolate from OKNP, one gorilla isolate from LLR and three human isolates that are fully identical and they are all very similar to SAdV-42.2. In addition, two other isolates from LLR gorillas had close similarity with SAdV-42.1; (ii) Clade 2: a gorilla AdV from OKNP with perfect similarity to HAdV-C K67-339 (LC504573); (iii) Clade 3: a gorilla AdV from LLR, closely related to *Gorilla beringei graueri* AdV-9 [36]. HAdV-C types had already been reported in free ranging gorillas from Republic of Congo [31] and Gabon [33], wild mountain (*Gorilla beringei beringei*) and Grauer’s (*Gorilla beringei graueri*) gorillas from Rwanda [36] and captive gorillas, as well as captive chimpanzees and bonobos [13]. According to Hoppe et al. [32] and other studies [13,33], it seems that all the lineages in HAdV-C are host specific. Nevertheless, we report here that both a gorilla AdV (G10) and human AdVs (Mbo025, 058 and Ibou01) are related to species HAdV-C (Figure 2), which is evidence for cross-species and potential zoonotic transmission. As a result, HAdV-C types could have a wider range of hosts rather than being strictly host species-specific. Wild gorillas and chimpanzees that do not necessarily come into daily contact with humans carry human-associated adenoviruses. The same was true for the local human population, including the eco-guards that carried simian viruses. This suggests that a kind of “jump” has occurred.

A HAdV-B type detected here in a Senegalese chimpanzee was close to SAdV-41.1 which was detected in a captive gorilla [13]. AdVs belonging to this species have been previously reported in chimpanzees and gorillas from the Republic of Congo [31], wild gorillas from Gabon [33] and both captive and wild gorillas and chimpanzees [13,32]. Wevers et al. [5] reported HAdV-B types in 53 gorillas and six chimpanzees and confirmed for wild individuals that members of HAdV-B widely infect great apes. However, viruses of the same host species strayed throughout the clade, and several subclades comprising AdVs of different hosts were visible [5]. They suggested that human HAdV-B originated in great apes. In our study, the HAdV-B type that was found in chimpanzees formed a separate lineage from the clade of HAdV-B.

Among human AdVs, HAdV-D is the largest and fastest growing species [5]. We detected HAdV-D members in the stool of humans living around the OKNP, thus confirming that the species HAdV-D originated in humans [32] and so far has been exclusively human specific. Nkogue et al. reported four different serotypes in a human population around a gorilla park, highlighting the diversity of AdVs circulating in humans [33]. Furthermore, it has been found that AdVs from wild chimpanzees clustered in HAdV-D and showed a 99% pairwise hexon nucleic acid identity with HAdV-15 and a 98% identity with HAdV-29 and -30 [5]. As a result of the co-habitation of eco-guards with NHPs, the transmission of these viruses from humans to NHPs could eventually lead to the extension of HAdV-D members in NHP communities. In our study, viral isolation was possible only in a human sample as the NHP samples were not fresh. AdV strain Mbo024 was identified as a HAdV-D member and was found to be almost identical to HAdV-25, thus confirming the PCR results. Unfortunately, it has not been possible to isolate other AdVs in humans or NHPs in order to better understand their genetic diversity and relationships. In addition to the degraded quality of the NHP samples, failures of the simian virus culture could be related to the lack of a suitable culture system, since the culture was performed using human cell lines.

Finally, our results show that AdVs are naturally present among African NHPs and the human communities living near them. Wild chimpanzees and gorillas of the same population carry a variety of AdV genotypes, suggesting that great apes are the origin of many HAdVs. These viruses are maintained by the exchange between ape individuals and potentially with human populations that live nearby. One of the strengths of this study was that we analyzed the fecal samples of different NHP species with different origins (North Africa and sub-Saharan Africa) as well as samples from humans living in contact with primates in order to evaluate the potential for zoonotic transmission. Moreover, the fact that while the target partial gene seemed to be linked even across species, one organism could be capable of hosting simian and human viruses and recombination events, is not highlighted here, but it is a possibility. We complement and enrich recent knowledge (e.g., [5,13,32]) which has suggested that HAdV evolution has been mainly governed by strong, long-term associations with their hominid hosts. AdV species (in particular HAdV-E) have been found to be represented in several primates, suggesting, most likely, cross-species transmission. Moreover, we found evidence of the zoonotic transmission of adenoviruses, particularly in HAdV-C members, in the Republic of Congo.

## 5. Conclusions

Our study reported a rich diversity of adenovirus types in HAdV-E, HAdV-C and HAdV-B among African apes and/or monkeys as well as indigenous human populations (HAdV-C and HAdV-D). AdV shedding was more prevalent in NHPs than in humans (infection rate was 3.3 folds higher in NHPs compared to humans). HAdV-E types were the most predominant ones in NHPs regardless of their origins, and they seemed to lack strict host specificity. The AdVs from NHPs that were detected here (especially AdV-C members) are common in NHPs and human populations living nearby, thus suggesting a zoonotic transmission, meaning that these viruses have switched hosts. This has been especially true for crossing the species barrier between different NHP species (especially in the case of the AdV-E members), between NHPs and humans, but it may also occur between humans, NHPs and other animal species.

## Figures and Tables

**Table 1:** PCR systems used in the present study.

PCR System	Target Gene	Name of Primer	Sequence	Fragment Length	Tm °C	Source
Extraction control	Phage T4	T4F	CCATCCATAGAGAAAATATCAGAACGA	-	60	[22]
		T4R	TAAATAATTCCTCTTTTCCCAGCG			
		T4probe	VIC-AACCAGTAATTTCATCTGCTTCTGATGTGAGGC			
Adenoviruses	Hexon	AQ1	GCCACGGTGGGGTTTCTAAACTT	-	60	[23]
		AQ2	GCCCCAGTGGTCTTACATGCACAT C			
		AP	6FAM-TGCACCAGACCCGGGCTCAGGTACTCCGA			
	DNA polymerase	Fw Outer	TGATGCGYTTCTTACCTYTGGTYTCCATGAG	≈1400 bps	58	[13]
		Rw Outer	AGTTYTACATGCTGGGCTCTTACCG			
		Fw inere	GTGACAAAGAGGCTGTCCGTGTCCCCGTA	≈250 bps	55	
		Rw inere	TCACGTGGCCTACACTTACAAGCCAATCAC			

**Tm:** annealing temperature.

**Table 2:** Prevalence of adenoviruses in African humans and non-human primates (NHPs).

Species	Origin	N	Positive	Negative	Prevalence (%)
Gorilla	Rep. of Congo	39	14	25	35.9
Chimpanzee	Senegal	48	26	22	54.2
Guinea baboon	Senegal	7	2	5	28.6
Hamadryas baboon	Djibouti	6	1	5	16.7
Green monkey	Senegal	4	0	4	0.0
Macaque	Algeria	69	19	50	27.5
Great apes	-	87	40	47	46.0
Monkeys	-	86	22	64	25.6
Total NHPs	-	173	62	111	35.8
Humans	Rep. of Congo	38	4	34	10.5

## Supplementary Materials

The following are available online at https://www.mdpi.com/1999-4915/12/6/657/s1, Table S1. Adenoviruses identified in the present study in African humans and NHPs (sequences of ≈250 bp DNA polymerase gene) and their identity with GenBank reference sequences; Table S2: Mbo024 gene annotation and comparison to other related *Human mastadenovirus D* types. Supp. Material S1: whole genome Mbo024.
