# Correction: Medkour et al. Adenovirus Infections in African Humans and Wild Non-Human Primates: Great Diversity and Cross-Species Transmission. *Viruses* 2020, *12*, 657

**DOI:** 10.3390/v18060601

**Published:** 2026-05-26

**Authors:** Hacène Medkour, Inestin Amona, Jean Akiana, Bernard Davoust, Idir Bitam, Anthony Levasseur, Mamadou Lamine Tall, Georges Diatta, Cheikh Sokhna, Raquel Adriana Hernandez-Aguilar, Amanda Barciela, Slim Gorsane, Bernard La Scola, Didier Raoult, Florence Fenollar, Oleg Mediannikov

**Affiliations:** 1 IHU Méditerranée Infection, 13385 Marseille CEDEX 05, France; hacenevet1990@yahoo.fr (H.M.); amoninestin@gmail.com (I.A.); bernard.davoust@gmail.com (B.D.); anthony.levasseur@univ-amu.fr (A.L.); laminetall30@gmail.com (M.L.T.); cheikh.sokhna@ird.fr (C.S.); bernard.la-scola@univ-amu.fr (B.L.S.); didier.raoult@gmail.com (D.R.); florence.fenollar@univ-amu.fr (F.F.); 2 Aix-Marseille University, IRD, AP-HM, Microbes, MEPHI, 13385 Marseille CEDEX 05, France; 3 PADESCA Laboratory, Veterinary Science Institute, University Constantine 1, El Khroub 25100, Algeria; 4 Aix-Marseille University, IRD, AP-HM, SSA, VITROME, 13385 Marseille CEDEX 05, France; idirbitam@gmail.com (I.B.); georges.diatta@ird.fr (G.D.); 5 Faculté des Sciences et Techniques, Université Marien NGOUABI, Brazzaville, Democratic Republic of the Congo; 6 Laboratoire National de Santé Publique, Brazzaville, Democratic Republic of the Congo; jakiana2000@yahoo.fr; 7 Superior School of Food Sciences and Food Industries, Algiers 16004, Algeria; 8 VITROME IRD 198, Campus IRD/UCAD, Hann les Maristes, Dakar, Senegal; 9 Department of Social Psychology and Quantitative Psychology, Faculty of Psychology, University of Barcelona, Passeig de la Vall d’Hebron 171, 08035 Barcelona, Spain; r.a.hernandez-aguilar@ibv.uio.no; 10 Jane Goodall Institute Spain and Senegal, Dindefelo Biological Station, Dindefelo, Kedougou, Senegal; amanda.b@janegoodall.es; 11 Direction Interarmées du Service de Santé des Armées des Forces Françaises Stationnées à Djibouti; slim.gorsane@intradef.gouv.fr

In the original publication [1], the sections titled “Institutional Review Board Statement” and “Informed Consent Statement” were not clearly defined. We have now added these two sections as follows:

**Institutional Review Board Statement**: Due to the nature of the study, which involved strictly non-invasive sample collection, and considering the regulatory context at the time of sample collection, formal approval from an Institutional Review Board or National Ethics Committee was not required. In Senegal in 2016, the study was conducted in accordance with the authorization granted by the Direction des Eaux, Forêts, Chasses et Conservation des Sols of the Republic of Senegal (No. 1914/DEF/DGF, 5 June 2016). In the Republic of Congo in 2015, in the absence of a National Ethics Committee at the time of sample collection, and following the requirements of the only competent authority, the Ministry of Health and Population, an official research authorization was obtained (No. 00208/MSP/CAB.15, 20 August 2015). In the Republic of Congo in 2017, the study was conducted in accordance with the authorization granted by the Ministry of Health and Population through an official Ordre de Mission (No. 000220/MSP-CAB-17, 23 October 2017).
**Informed Consent Statement**: Verbal informed consent was obtained from the participants. Verbal consent was obtained rather than written because in rural areas, obtaining written informed consent is constrained by sociocultural, educational, and linguistic factors. Low literacy levels, particularly among adult and elderly populations, limit participants’ ability to meaningfully engage with written consent forms. In addition, written documents are often perceived as administrative or legal instruments, which may generate mistrust or reluctance to participate. Linguistic diversity further complicates the use of written consent, as study documents are typically drafted in French while participants primarily communicate in local languages. In this context, oral informed consent was obtained from all participants following a clear explanation of the study objectives, procedures, potential benefits, and risks in the participant’s native language. Participation was voluntary, refusal entailed no consequences, and no personally identifiable information was collected. When appropriate, consent was obtained in the presence of a witness.

The authors state that the scientific conclusions are unaffected. This correction was approved by the Academic Editor. The original publication has also been updated.
