# Supplementary material for: Adenovirus Infections in African Humans and Wild Non-Human Primates: Great Diversity and Cross-Species Transmission
Source: Viruses. 2020 Jun 18;12(6):657. doi: 10.3390/v12060657 (PMC7354429; doi:10.3390/v12060657)
Supplement: Supplementary file 1 [file viruses-12-00657-s001.zip › viruses-803616-SI/Table S1.pdf]

Table S1. Adenoviruses identified in the present study in African humans and NHPs (sequences of ≈250 bps DNA polymerase gene) and their identity with GenBank reference sequences.

Code	Species	Origine	Best Blastn GenBank	% Identity	% Cover	Type
**GC01**	*Macaca sylvanus*	Algeria	FJ025922.1 SAdV38	98	100	E
**GC08**	*Macaca sylvanus*	Algeria	FJ025922.1 SAdV38	98	100	E
**GC11**	*Macaca sylvanus*	Algeria	FJ025922.1 SAdV38	100	100	E
**RS04**	*Macaca sylvanus*	Algeria	FJ025922.1 SAdV38	100	99	E
**RS13**	*Macaca sylvanus*	Algeria	FJ025922.1 SAdV38	100	99	E
**TE01**	*Macaca sylvanus*	Algeria	FJ025922.1 SAdV38	99.5	99	E
**TE02**	*Macaca sylvanus*	Algeria	FJ025922.1 SAdV38	100	99	E
**G1**	*Gorilla gorilla*	Rep. Congo	FJ025922.1 SAdV38	100	100	E
**G11**	*Gorilla gorilla*	Rep. Congo	KC702817.1 Gorilla b. graueri AdV 8 isolate GC45	97.6	100	C
**CHS03**	*Pan troglodytes*	Senegal	FJ025927.1 SAdV41.2	97.1	100	B
**CHS11**	*Pan troglodytes*	Senegal	FJ025922.1 SAdV38	98.7	100	E
**CHS18**	*Pan troglodytes*	Senegal	FJ025922.1 SAdV38	100	100	E
**CHS20**	*Pan troglodytes*	Senegal	FJ025922.1 SAdV38	99.5	100	E
**CHS28**	*Pan troglodytes*	Senegal	FJ025922.1 SAdV38	100	100	E
**CHS43**	*Pan troglodytes*	Senegal	FJ025922.1 SAdV38	100	100	E
**CHS25**	*Pan troglodytes*	Senegal	FJ025922.1 SAdV38	100	100	E
**CHS27**	*Pan troglodytes*	Senegal	FJ025922.1 SAdV38	100	100	E
**CHS32**	*Pan troglodytes*	Senegal	FJ025922.1 SAdV38	100	100	E
**CHS44**	*Pan troglodytes*	Senegal	FJ025922.1 SAdV38	100	100	E
**Bab 1**	*Papio hamadryas*	Djibouti	FJ025922.1 SAdV38	100	100	E
**G06**	*Gorilla gorilla*	Rep. Congo	FJ025903.1 SAdV42.1	99.5	100	C
**G10**	*Gorilla gorilla*	Rep. Congo	LC504573.1 HAdV-C K67-339	100	100	C
**G02A**	*Gorilla gorilla*	Rep. Congo	FJ025903.1 SAdV42.1	99.4	99	C
**G04A**	*Gorilla gorilla*	Rep. Congo	FJ025903.1 SAdV42.1	100	100	C
**G07A**	*Gorilla gorilla*	Rep. Congo	FJ025903.1 SAdV42.1	99.5	100	C
**Ibou01A**	*Human*	Rep. Congo	FJ025903.1 SAdV42.1	99.5	100	C

**Mbou25**	*Human*	Rep. Congo	FJ025903.1 SAdV42.1	99.5	100	C
**Mbou058**	*Human*	Rep. Congo	FJ025903.1 SAdV42.1	99.5	100	C
**Mbou024**	*Human*	Rep. Congo	KF268200.1 HAdV-D strain human/USA/UFL/2005[P-NEW/H45/F-NEW]	98	100	D
