# Supplementary material for: Adenovirus Infections in African Humans and Wild Non-Human Primates: Great Diversity and Cross-Species Transmission
Source: Viruses. 2020 Jun 18;12(6):657. doi: 10.3390/v12060657 (PMC7354429; doi:10.3390/v12060657)
Supplement: Supplementary file 1 [file viruses-12-00657-s001.zip › viruses-803616-SI/Table S2..pdf]

**Table S2.** Mbou024gene annotation and comparison to other related *Human mastadenovirus D* types

| | | Amino acid identity (%) | | | | | | | | | |
|---|---|---|---|---|---|---|---|---|---|---|---|
| **Region** | **Genome location** | **HAdV-D (KF268332.1)** | **HAdV-13 (JN226747.1)** | **HAdV-25 (JN226752.1)** | **HAdV-26 (EF153474.1)** | **HAdV-36 (GQ384080.1)** | **HAdV-42 (JN226761.1)** | **HAdV-47 (JN226757.1)** | **HAdV-49 (DQ393829.1** | **HAdV-65 (AP012285.1)** | **HAdV -67 (AP012302.1)** |
| E1A | 380-1236 | 91.64 | 94.06 | 88.15 | 92.89 | 92.78 | 90.01 | 95.92 | 89.66 | 88.62 | 96.27 |
| E1B | 1389-3180 | 94.76 | 93.78 | 94.98 | 94.75 | 94.64 | 94.20 | 96.48 | 95.31 | 94.75 | 94.36 |
| pIX | 3268-3659 | 98.77 | 98.22 | 98.83 | 98.71 | 98.34 | 98.03 | 98.15 | 98.09 | 98.59 | 98.71 |
| pIVa2 | 3712-5336 | 98.77 | 98.22 | 98.83 | 98.77 | 98.34 | 98.03 | 98.15 | 98.09 | 98.58 | 98.77 |
| E2B | 4815-8193 | 97.34 | 97.25 | 97.40 | 97.25 | 97.28 | 97.34 | 97.52 | 97.28 | 97.04 | 97.07 |
| | 8199-9982 | 97.93 | 97.83 | 97.88 | 97.77 | 98.10 | 97.99 | 98.27 | 98.10 | 97.32 | 97.71 |
| | 10046-10621 | 97.92 | 98.79 | 97.92 | 97.75 | 97.75 | 98.09 | 97.57 | 97.93 | 97.40 | 97.71 |
| | 10733-11129 | 98.74 | 98.49 | 97.73 | 97.98 | 98.24 | 98.49 | 98.74 | 97.98 | 97.48 | 97.48 |
| | 11224-12831 | 98.32 | 98.33 | 97.89 | 97.83 | 98.26 | 97.96 | 98.02 | 97.58 | 97.02 | 97.03 |
| pTP | 8227-9982 | 98.35 | 98.29 | 98.29 | 98.18 | 98.52 | 98.41 | 98.69 | 98.52 | 97.72 | 98.12 |
| | 10046-10093 | 97.92 | 97.92 | 97.92 | 97.92 | | 97.92 | 97.92 | 97.92 | 97.92 | |
| L1 | 10540-10621 | 97.56 | 100.00 | 100.00 | 100.00 | 98.78 | 100.00 | 100.00 | 100.00 | 98.78 | 98.78 |
| | 10733-11129 | 98.74 | 98.49 | 97.73 | 97.98 | 98.24 | 98.49 | 98.74 | 97.98 | 97.48 | 97.48 |
| | 11224-13020 | 98.00 | 98.06 | 97.10 | 97.16 | 97.38 | 96.89 | 97.78 | 96.72 | 96.39 | 96.39 |
| | 13012-13424 | 96.66 | 96.57 | 96.45 | 96.69 | 96.32 | 95.86 | 96.57 | 98.45 | 96.45 | 96.33 |
| L2 | 13475-14313 | 96.66 | 96.57 | 96.45 | 96.69 | 96.32 | 95.86 | 96.57 | 98.45 | 96.45 | 96.33 |
| | 14486-15085 | 98.83 | 97.34 | 98.28 | 97.77 | 99.14 | 99.31 | 98.28 | 99.17 | 98.00 | 97.79 |
| | 16581-16904 | 99.69 | 99.07 | 99.38 | 99.07 | 100.00 | 99.38 | 98.77 | 99.69 | 99.38 | 99.07 |
| L3 | 16960-17068 | 100.00 | 100.00 | 100.00 | 100.00 | 100.00 | 100.00 | 100.00 | 100.00 | 100.00 | 100.00 |
| | 17563-17984 | 99.29 | 99.29 | 99.53 | 99.29 | 99.29 | 99.53 | 99.76 | 99.53 | 99.53 | 99.76 |
| | 18042-18435 | 83.79 | 78.00 | 80.72 | 76.27 | 79.95 | 80.69 | 81.93 | 81.36 | 76.85 | 78.52 |
| | 18533-19283 | 87.88 | 86.29 | 85.68 | 85.73 | 86.22 | 85.88 | 87.70 | 86.31 | 86.54 | 86.31 |
| | 19325-21187 | 97.64 | 97.91 | 97.69 | 97.85 | 97.26 | 97.26 | 98.01 | 97.85 | 97.31 | 97.80 |
| E2A | 21242-22711 | 95.33 | 97.09 | 95.73 | 95.73 | 95.19 | 94.39 | 96.21 | 95.67 | 94.73 | 94.85 |
| L4 | 22728-22845 | 88.10 | 90.77 | 89.68 | 89.68 | 89.68 | 89.68 | 89.68 | 89.68 | 88.10 | 86.05 |

| | | | | | | | | | | | |
|---|---|---|---|---|---|---|---|---|---|---|---|
| | 22835-23293 | 95.86 | 99.13 | 97.84 | 97.19 | 98.27 | 98.05 | 99.13 | 97.19 | 96.51 | 95.64 |
| | 23369-26089 | 96.88 | 97.87 | 96.33 | 96.70 | 96.00 | 96.11 | 98.13 | 97.10 | 96.11 | 96.29 |
| E3 | 26090-28741 | 95.21 | 94.86 | 87.75 | 88.43 | 90.62 | 86.98 | 91.95 | 95.57 | 86.82 | 88.28 |
| | 29139-30194 | 93.82 | 95.68 | 91.85 | 92.43 | 92.29 | 93.63 | 92.98 | 94.25 | 92.39 | 91.66 |
| | 30298-30680 | 99.22 | 98.4 | 97.91 | 98.96 | 98.43 | 99.22 | 98.69 | 99.21 | 98.69 | 98.69 |
| U | 30760-30909 | 98.00 | 97.33 | 94.67 | 98.00 | 98.00 | 96.67 | 98.00 | 98.00 | 97.33 | 96.67 |
| L5 | 30925-31147 | 94.95 | 95.52 | 95.00 | 96.12 | 97.14 | 94.62 | 96.86 | 95.93 | 93.27 | 95.00 |
| E4 | 32061-34636 | 94.41 | 94.14 | 94.17 | 94.14 | 95.89 | 94.92 | 96.82 | 94.64 | 92.93 | 94.41 |
